# Effect of Teriparatide on Bone Mineral Density and Bone Markers in Real-Life: Argentine Experience

**DOI:** 10.1155/2023/9355672

**Published:** 2023-01-13

**Authors:** Rodolfo Guelman, Ariel Sánchez, Mariela Varsavsky, Lucas R. Brun, María Laura García, Marcelo Sarli, Rey Paula, Vanina Farias, María Belén Zanchetta, Evangelina Giacoia, Helena Salerni, Laura Maffei, Valeria Premrou, Beatriz Oliveri, María Lorena Brance, Magdalena Pavlove, Silvia Karlsbrum, María Silvia Larroudé, Pablo René Costanzo

**Affiliations:** ^1^Servicio de Endocrinología y Medicina Nuclear, Hospital Italiano de Buenos Aires, Buenos Aires, Argentina; ^2^Centro de Endocrinología, Rosario, Argentina; ^3^CONICET, Rosario, Argentina; ^4^Laboratorio de Biología Ósea, Fac Cs Médicas, Universidad Nacional de Rosario, Rosario, Argentina; ^5^Sanatorio Julio Méndez, Buenos Aires, Argentina; ^6^Instituto de Investigaciones Metabólicas, Universidad del Salvador, Buenos Aires, Argentina; ^7^Servicio de Endocrinología y Metabolismo, Hospital Posadas, Buenos Aires, Argentina; ^8^Consultorios de Investigación Clínica Endocrinológica y del Metabolismo Óseo (CICEMO), Buenos Aires, Argentina; ^9^Consultorios Asociados de Endocrinología Dra. Laura Maffei, Buenos Aires, Argentina; ^10^Mautalen Salud e Investigación, Buenos Aires, Argentina; ^11^Reumatología y Enfermedades Óseas, Rosario, Argentina; ^12^Hospital Durand, Buenos Aires, Argentina; ^13^Centro Rossi, Buenos Aires, Argentina

## Abstract

**Purpose:**

To evaluate the effect of teriparatide (TPTD) on bone mineral density (BMD) and bone markers under clinical practice conditions. To assess whether the results in real-life match those published in clinical trials.

**Methods:**

Cross-sectional study of postmenopausal women treated with TPTD for at least 12 months.

**Results:**

264 patients were included in the study. Main characteristics are as follows: age: 68.7 ± 10.2 years, previous fractures: 57.6%, and previously treated with antiresorptive (AR-prior): 79%. All bone turnover markers studied significantly increased after 6 months. CTX and BGP remained high up to 24 months, but total and bone alkaline phosphatase returned to basal values at month 18. There was a significant increase in lumbar spine (LS) BMD after 6 months (+6.2%), with a maximum peak at 24 months (+13%). Femoral neck (FN) and total hip (TH) BMD showed a significant increase later than LS (just at month 12), reaching a maximum peak at month 24 (FN + 7.9% and TH + 5.5%). A significant increase in LS BMD was found from month 6 to month 24 compared to basal in both AR-naïve, and AR-prior patients (+16.7% and +10.5%, respectively), without significant differences between the two groups. Comparable results were found in FN and TH BMD. *Main conclusions*. As reported in real-life clinical studies, treatment of osteoporotic postmenopausal women with TPTD induced a significant increase in bone turnover markers from month 6 onward and an increase in BMD from months 6–12 with continuous gain up to month 24. The real-life results of our study matched the results of randomized clinical trials. In addition, TPTD induced an increase in BMD, regardless of the previous use of AR.

## 1. Introduction

Osteoporosis is a chronic condition characterized by lower bone mass and bone microarchitecture deterioration, which compromises bone strength and increases fragility fractures. Currently available treatments for osteoporosis are antiresorptive (AR) or anti-catabolic medications, such as bisphosphonates (BP), denosumab (Dmab), oestrogens, and selective oestrogen receptor modulators, as well as bone-forming agents, such as parathyroid hormone (PTH_1-84_ or its fragment PTH_1-34_) and abaloparatide. Another treatment recently approved in several countries includes romosozumab.

Teriparatide (TPTD) treatment stimulates bone formation, increasing bone remodeling, trabecular connectivity, and cortical thickness [1–4]. TPTD is then considered a potent bone anabolic treatment, improving bone biomechanics and reducing vertebral and nonvertebral fracture risk in postmenopausal women. It is also effective and approved for men with osteoporosis and glucocorticoid-induced osteoporosis [5–8].

In Argentina, TPTD is approved as an initial treatment for severe osteoporosis, very low bone mass (T-score −3 SD), and previous fragility fracture in patients with remarkably high or imminent risk of fracture, or in cases of intolerance or failure of other treatments (intratreatment fracture or decrease in bone mineral density). Although patients are not prescribed TPTD when necessary for several reasons, such as affordability and insufficient medical knowledge, among others, the vast majority are previously prescribed AR agents.

Treating patients with BP or Dmab before TPTD has been reported to induce less increase in bone mineral density (BMD) and less anti-fracture efficacy, especially at the hip [9–11].

Clinical randomized trials are the gold standard to demonstrate treatment efficacy, but observational trials based on daily clinical practice have enlarged efficacy and safety data in the real world and provided additional information. It is estimated that 80% of patients following osteoporosis treatment would not meet the inclusion criteria to participate in clinical trials, even when these patients tend to be more compliant [12, 13].

This study aimed at evaluating the effect of TPTD on BMD and biochemical markers of bone turnover under clinical practice conditions in patients treated at centers specialized in bone metabolism. Additionally, the effect of TPTD on BMD in AR-naïve patients was compared with the effects on patients previously treated with AR (AR-prior).

## 2. Patients and Methods

This is a retrospective, cross-sectional, and multicenter study (11 centers in Argentina) in 264 postmenopausal women treated with TPTD at least for 12 months between 2006 and 2018. All women had either a T-score of less than −2.5 at the hip or lumbar spine or a T-score of less than −2.0 plus other risk factors for fracture. As inclusion criteria, we also considered that the patients had performed a BMD at least basal after 12 months. All patients simultaneously received calcium (at least 1000 mg/day) and vitamin D (at least 800 IU/day).

In addition, patients were analyzed considering the previous use of AR and were divided into AR-naïve (*n* = 56) and AR-prior (*n* = 208). The use of TPTD was considered after a lack of response to treatment with BP (38.3%), multiple fractures (17.8%), extremely low BMD (16.3%), or combinations thereof (24.6%). The remaining 3% were due to atypical fractures (*n* = 6) and delays in fracture healing (*n* = 2).

Baseline characteristics, biochemical parameters, and BMD were obtained from medical records. Serum calcium, phosphate, magnesium, uric acid, total phosphate (tAP), and bone alkaline phosphatase (bAP) levels were measured spectrophotometrically. Urinary calcium was measured in 24 h urine. Serum parathyroid hormone (PTH), total serum 25-hydroxyvitamin D levels (25OHD), and urinary deoxypyridinoline (Dpyr) were measured by chemiluminescence assay. Osteocalcin (BGP) and serum carboxy-terminal crosslinking telopeptide of type I collagen (CTX) were measured by electrochemiluminescence assay. Bone markers were analyzed at 0 (basal), 3, 6, 12, 18, and 24 months under TPTD treatment.

BMD (g/cm^2^) was measured by dual-energy X-ray absorptiometry (DXA) with the GE Lunar Prodigy (GE Lunar, USA) at the lumbar spine (LS, L1-L4), femoral neck (FN), and total hip (TH) at 0 (basal), 6, 12, 18, and 24 months under TPTD treatment. Scans were performed according to the recommendations provided by the manufacturer, and the coefficient of variation was less than 2% at all centers [14]. Clinical vertebral fractures were studied with radiography, tomography, or magnetic resonance imaging.

The study was conducted in accordance with the Helsinki declaration. Each participant was identified by a number to keep their identity confidential.

## 3. Data Analysis

Kolmogorov–Smirnov test for normality was used to assess the distribution of data, and the test was used as appropriate. Student's T-test or Mann–Whitney test was used to compare the two groups. A Wilcoxon signed-rank test was used to compare paired data. Data are expressed as the mean ± SD or mean ± SEM. Differences were considered significant if *p* < 0.05. Statistical analyses were performed with GraphPad Prism 5.01 (GraphPad, San Diego, USA).

## 4. Results

### 4.1. Baseline Clinical Characteristics

Medical records of 264 postmenopausal women treated with TPTD at least for 12 months were analyzed. In 71.2% (*n* = 188) and 31.4% (*n* = 83), the TPTD treatment was received at least during 18 and 24 months, respectively. Baseline biochemical parameters and BMD are shown in Table 1. The main characteristics of the study population were age 68.7 ± 10.2 years (range: 43–101), body mass index (BMI): 24.7 ± 4.4 kg/m^2^, age of menopause: 48.0 ± 4.7 years, previous fractures: 57.6% (median of vertebral fractures: 2 (range 1–9) and nonvertebral fractures: 1 (range 1–5)).

Almost 79% (208/264) of the patients had used BP or Dmab treatment previously with a treatment mean duration of 5.9 ± 3.6 years: 65.4% of patients who used BP (*n* = 136) received only one BP, 25.9% (*n* = 54) were switched to another BP, and 8.7% (*n* = 18) were switched to Dmab (treatment duration 1.6 ± 0.7 years) before TPTD. In patients who received only one BP (65.4%), 82.4% were oral BP (59.8% alendronate, 30.8% ibandronate, and 9.4% risedronate), and 17.6% were intravenous (mainly zoledronate). In patients who were switched to a second BP before TPTD, 42.1% were switched to another oral BP, 45.6% were switched from oral BP to intravenous BP, and 12.3% were switched from intravenous BP to oral BP.

### 4.2. Changes in BMD after TPTD Treatment

**Figure 1:**
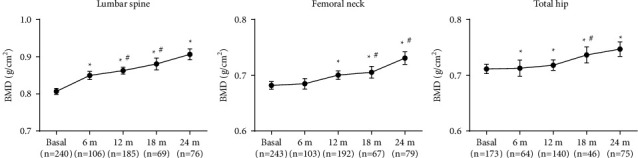
Changes in bone mineral density during treatment with teriparatide. Wilcoxon signed-rank test. Mean ± SEM. ^*∗*^Indicates significant differences vs. basal (*p* < 0.05). ^#^Indicates significant differences vs. previous (*p* < 0.05).

After TPTD treatment, there was a significant increase in LS BMD from month 6 (+5.3%) with a maximum peak at month 24. From baseline to end-point, TPTD increased LS BMD by 12.3% (*p* < 0.0001). TH and FN BMD showed a significant increase from month 6 and 12, respectively, reaching a maximum peak at month 24 (TH +5.0%; FN: +7.2%; *p* < 0.0001) (Figure 1).

### 4.3. Changes in Biochemical Parameters after the TPTD Treatment

**Figure 2:**
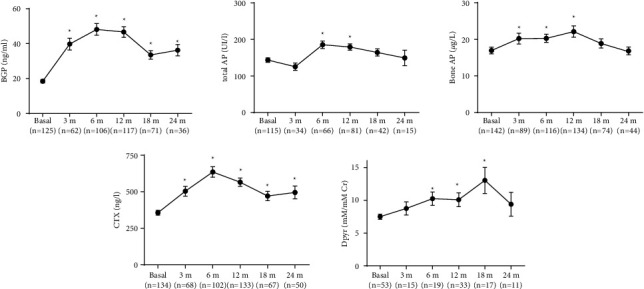
Changes in bone formation and resorption markers during treatment with teriparatide. Wilcoxon signed-rank test. Mean ± SEM. ^*∗*^Indicates significant differences vs. basal (*p* < 0.05).

tAP and bAP significantly increased from month 6 and 3, respectively, returning to basal values at month 18. While BGP significantly increased from month 3 and remained increased at month 24 with a maximum peak at month 6 (+160.8%). Dpyr significantly increased from month 6, returning to basal values in month 24, while s-CTX significantly increased from month 3 and remained high up to month 24 with a maximum peak at month 6 (+78.1%) (Figure 2).

PTH decreased significantly at month 6 and returned to baseline values at month 18. A sustained increase from month 3 to 24 was found for serum and urinary calcium, without clinical hypercalcemia or nephrolithiasis. No significant differences were found in serum phosphate and 25OHD during treatment. Magnesium significantly decreased from month 3 until month 24 without hypomagnesemia, while uric acid showed an inverse pattern of behavior (Table 2).

### 4.4. Analysis of Patients According to AR before the Treatment with TPTD

Patients were also analyzed considering the previous use of AR : AR-naïve (*n* = 56, 21.2%) and AR-prior (*n* = 208, 78.8%). The duration of previous AR treatment was 5.9 ± 3.6 years. There were no significant differences in BMI, years of menopause, vertebral and nonvertebral fractures, TPTD treatment period, and basal serum or urinary calcium values, PTH, and 25OHD between the AR-naïve and AR-prior groups. In addition, no differences in basal LS, FN, and TH BMD were observed (Table 1). Age (AR-naïve: 66.2 ± 11.6 and AR-prior: 69.4 ± 9.7 years; *p*=0.0320) varied between groups. As expected, basal BGP (AR-naïve: 22.8 ± 12.6 ng/ml; AR-prior: 17.4 ± 10.1 ng/ml; *p*=0.0393) and s-CTX (AR-naïve: 438.1 ± 187.0 ng/l; AR-prior: 334.4 ± 187.4 ng/l; *p*=0.0044) were significantly lower in the AR-prior group. However, tAP, bAP, and Dpyr showed no significant differences between groups.

**Figure 3:**
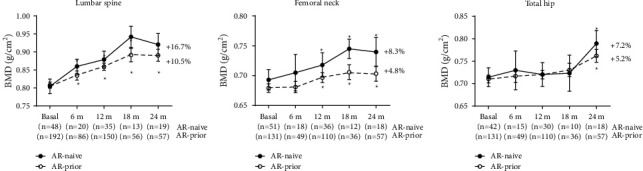
Changes in BMD in antiresorptive (AR)-naïve vs. AR-prior patients. Wilcoxon signed-rank test. Mean ± SEM. ^*∗*^Indicates significant differences vs. basal.

The increases in LS and FN BMD after TPTD treatment were also found in AR-naïve, and AR-prior patients from month 6 and 12, respectively, reaching a maximum peak at month 18 (LS AR-naïve +21.6%; LS AR-prior +10.5%; FN AR-naïve +10.5%; FN AR-prior +4.8; *p* < 0.01). Despite higher values in AR-naïve, no statistical differences were observed (*p* > 0.05) (Figure 3). TH BMD also increased in AR-naïve patients (+7.2% at month 24, *p*=0.0009), while in AR-prior patients there was a significant increase from month 4, reaching a maximum peak at month 24 (+5.2, *p*  < 0.0001).

The subgroup of AR-prior patients who also received Dmab (*n* = 18) had no differences compared to the whole AR-prior group. LS BMD significantly increased at month 6 (+6.9%) and month 12 (+8.0%), while FN and TH significantly increased at month 12 (+2.5% and 2.6%, respectively). Additional analyses at months 18 and 24 were not performed due to an insufficient number of patients.

## 5. Discussion

TPTD has both a direct action on osteoblast receptors and a reduced effect on sclerostin production by osteocytes, causing an increase in proliferation and differentiation of osteoblast precursors through canonical Wnt signalling. This process, together with the stimulation of osteoblast function, led to increases in bone volume and a substantial proportion of new bone matrix in the trabecular and endocortical surfaces [15]. Further, TPTD also increased the production of RANK ligands by osteoblasts, resulting in osteoclast activation and, consequently, bone resorption.

In this real-life study, we observed an early increase in bone formation markers. BGP significantly increased from month 3 and remained over the 24 months of treatment. tAP and bAP increased from month 6 and 3, respectively, returning to basal values at month 18. Bone resorption markers also increased early: s-CTX significantly increased from month 3 and remained high up to month 24; Dpyr significantly increased from month 6, returning to basal values at month 18. The increase in bone formation markers was proportionally higher than the increase in bone resorption markers (BGP + 160.8% and s-CTX + 78.1%). Many studies show that P1NP is a bone formation marker that increases earlier and, therefore, is the most useful marker to assess TPTD action. This marker is not yet available in Argentina for clinical practice; thus, it was not included in our study [16, 17]. In said studies, P1NP rises early by 150%–300% from the basal values, similar to our results with BGP in real-life observation [18–21]. The s-CTX percentage of increase and its curve of rise in this study were similarly observed in other randomized trials [17, 21–23]. To our knowledge, there are no studies on bone turnover markers in real-life patients to be compared with our results. BGP was the bone marker that had shown the highest increase since the early months of the study and remained high during the 24 months of treatment. This might be useful to prove compliance and to assume treatment response before densitometric measurements.

Earlier and higher rise in bone formation than resorption allows for a significant gain in bone mass, especially in vertebrae with positive changes in microarchitecture, and consequently, in trabecular resistance [22]. As bone resorption increases, cortical porosity also increases, causing apparent detrimental changes in areal bone density in cortical regions, such as the hip and radius, thus explaining that bone mass in those areas may decrease when measured with DXA.

Based on medical literature, we observed a greater increase in LS BMD, significantly higher from month 6 (+5.3%) with a peak at month 24 (+12.3%), similar to data already published [5, 21, 23, 24]. The major increase observed in month 24 is similar to that reported in randomized control trials (Neer et al.) in 1637 patients, where an increase close to 10% was noted in month 21; in the EUROFORS study, the increase was 11.2% [5, 24].

TH and FN showed a significant increase from months 6 and 12, respectively, reaching a peak at month 24 (TH +5.0% and FN +7.2%). In randomized trials, hip BMD gain was also lower than LS, but in an even smaller proportion than in our study: +3% in the pivotal study and +4.2% in EUROFORS [5, 24]. Patients included in this study differ from those in the randomized trial because we also included patients with two or more fragility fractures and not only with vertebral fractures like those in the randomized trials. Other real-life studies assessed the risk of fracture, but they did not include data from BMD or bone markers in comparison with our study [1–4]. For the reasons explained above, the hip BMD increase might not be the same as that reported in other clinical trials. Such differences in daily practice might be possible if compared to randomized controlled clinical trials [5]. Observational studies would provide valuable additional information for further clinical trial conclusions.

There was a difference between patients in our study and the randomized trial patients since we also included patients with 2 or more fragility fractures (not only vertebral fractures like those in the randomized trials). Other real-life studies assessed the risk of fracture, but they did not take into account BMD or bone markers in comparison with our study [24–27]. For the reasons explained above, the hip BMD increase may not be the same as that reported in other clinical trials. Such differences in daily practice might be possible when compared to randomized controlled clinical trials [28]. Observational studies would provide valuable additional information for further clinical trial conclusions.

There is evidence that TPTD treatment responses may be different in those patients previously treated with AR vs. naïve patients [12]. AR reduces bone turnover, preventing bone tissue repair and causing its aging and hypermineralization. Newly formed bone tissue from anabolic therapy is less mineralized than older bone tissue. As it is widely known, DXA measures mineralized bone; thus, it may underestimate TPTD bone mass changes, overestimating those produced by AR [2, 29, 30].

The EUROFORS study included a cohort of women previously treated with BP who switched to TPTD for 24 months; those patients had an increase of 10.2% versus 13.1% without previous treatment. This is like our results: 10.5% vs. 16.7% [31]. This study also showed lesser increases of BMD in TH and FN than in LS, as well as less response capacity in those previously treated with AR vs. naïve patients (TH: 3.8% vs. 2.35%, FN: 4.8% vs. 3.9%). Similarly, we also found less gain in BMD in AR-prior (TH 7.2% vs. 5.2%, FN: 8.3% vs. 4.8%). Compared with this study, our results showed more gains in TH and FN BMD, but they were not significant. These differences between studies may be due to the size of our sample, which was smaller, in addition to the fact that we conducted a real-life study with a smaller number of DXA measurements which may lead to these statistical differences. A recent report, Lyu et al., with real-life data, analyzed patients who, after a median of 7 years under BP treatment, were switched to TPTD (*n* = 110) or Dmab (*n* = 105). Those on TPTD reduced hip BMD values to less than basal values in the first 12 months and then regained up to basal values, whereas those on Dmab did not reach the expected gain. We did not observe a decrease in hip BMD in AR-prior while on TPTD [32].

Sequential treatment study results suggest that TPTD as the first drug followed by AR [11, 17, 18, 33] is the ideal combination to obtain major gains in bone mass, as observed in our real-life study. Most of our patients, despite the presence of multiple fractures and very high risk, had been previously treated with AR for at least two years, but as with real-life patients, they were not always referred to specialists, and the concept of best sequential treatment was not well known.

Adverse events were reported in our study, such as small increases in serum calcium, serum uric acid, and 24 h urinary calcium, with a small decrease in serum magnesium; none of them were clinically significant and were similar to those found in the pivotal TPTD studies [5]. This could suggest that these parameters should be monitored or instanced during teriparatide treatment and eventually magnesium supplements are to be prescribed when its deficiency appears clinically relevant.

The limitations of our study are those of real-life evaluation since DXA and bone markers were not measured at a single center, even though the same methods were used. Not all patients had all the DXA or bone marker measurements. TPTD compliance was not evaluated. AR-naïve patients were fewer in number than AR-prior patients, losing some statistical value. Most of the AR-prior postmenopausal women had been switched to TPTD because of the poor clinical response to AR. The fracture outcome was not reported because this study was a retrospective real-life study, and not all patients had a spine X-ray to evaluate the morphometric fractures at the end of the treatment with teriparatide. Since we conducted a retrospective study, we were unable to collect exact information about dietary calcium intake and ongoing glucocorticoid treatment.

## 6. Conclusion

As reported in clinical studies, treatment of osteoporotic postmenopausal women with TPTD in real life induced a significant increase in bone turnover markers from month 6 on, with a higher impact on BGP and s-CTX markers, and an increase in the lumbar spine, femoral neck, and total hip BMD from months 6–12 with continuous gain up after 24 months. This increase was earlier and higher in the lumbar spine. In addition, TPTD in real-life induced an increase in bone turnover markers and BMD, regardless of the previous use of AR, although this was less evident in those who previously used AR. The fact that our biochemical results showed a safety profile like the pivotal studies should be highlighted.

## Figures and Tables

**Table 1:** Baseline biochemical parameters and BMD in the whole group of patients under teriparatide, and separately, in those with (AR-prior) and without (AR-naïve) previous antiresorptive treatment.

Basal characteristics		Whole group (*n* = 264)	AR-naïve (*n* = 56)	AR-prior (*n* = 208)
Age (years)		68.7 ± 10.2	66.2 ± 11.6	69.4 ± 9.7^#^
Body mass index (kg/m^2^)		24.7 ± 4.3	25.8 ± 5.4	24.4 ± 4.1
Serum calcium (mg/dL) (n. v: 8.5–10.5)		9.5 ± 0.4	9.5 ± 0.4	9.5 ± 0.5
Urinary calcium (mg/24 h)	149.8 ± 66.1		159.8 ± 74.5	147.1 ± 63.6
Calcium/creatinine (mg/mg)	0.2 ± 0.1		0.2 ± 0.1	0.2 ± 0.1
Serum phosphate (mg/dL) (n. v: 2.5–4.5)	3.8 ± 0.5		3.6 ± 0.5	3.8 ± 0.5^#^
Serum magnesium (mg/dL) (n. v: 1.9–2.3)	2.1 ± 0.2		2.1 ± 0.2	2.0 ± 0.2
Serum uric acid (mg/dL) (n. v: 2.4–6)	4.4 ± 1.0		4.5 ± 1.0	4.3 ± 1.0
25 (OH) vitamin D (ng/mL)	33.6 ± 12.7		30.6 ± 10.4	34.4 ± 13.0
PTH (pg/mL) (n. v: 10–65)	45.0 ± 18.8		43.5 ± 15.7	44.9 ± 19.8
Total alkaline phosphatase (IU/L) (n. v: <270)	145.3 ± 67.3		142.7 ± 67.6	143.7 ± 67.7
Bone alkaline phosphatase (*μ*g/L) (n.v: 5.2–24.4)	16.9 ± 10.7		24.2 ± 16.5	21.2 ± 14.5
Osteocalcin (ng/mL) (n. v: 11–43)	18.4 ± 10.8		22.8 ± 12.6	17.4 ± 10.1^#^
Urine deoxypyridinoline (nM/mM Cr) (n. v: 3–7.4)	7.4 ± 2.9		7.6 ± 1.4	7.5 ± 3.1
s-CTX (ng/ml) (n. v: 40–590)	356.80 ± 191.5		438.1 ± 187.0	334.4 ± 187.4^#^
Lumbar spine BMD (g/cm^2^; T-score)	0.807 ± 0.125; −3.3 ± 0.9		0.804 ± 0.141	0.807 ± 0.121
Femoral neck BMD (g/cm^2^; T-score)	0.682 ± 0.106; −2.5 ± 1.0		0.693 ± 0.123	0.679 ± 0.101
Total hip BMD (g/cm^2^; T-score)	0.711 ± 0.109; −2.5 ± 1.0		0.714 ± 0.135	0.710 ± 0.099

Mean ± SD. ^#^Indicates significant differences vs. AR-naïve.

**Table 2:** Changes in biochemical parameters during treatment with teriparatide.

	Basal	6 months	12 months	18 months	24 months
PTH (pg/mL)	44.5 ± 18.9 (*n* = 168)	36.5 ± 19.2^*∗*^(*n* = 62)	37.5 ± 19.0^*∗*^(*n* = 79)	38.8 ± 18.6 (*n* = 35)	42.9 ± 19.6 (*n* = 39)
Serum calcium (mg/dL)	9.5 ± 0.5 (*n* = 245)	9.8 ± 0.5^*∗*^(*n* = 168)	9.6 ± 0.5^*∗*^(*n* = 198)	9.7 ± 0.5^*∗*^(*n* = 103)	9.7 ± 0.5^*∗*^(*n* = 72)
Urinary calcium (mg/24 h)	149.8 ± 66.1 (*n* = 191)	199.7 ± 106.2^*∗*^(*n* = 110)	184.3 ± 117.5^*∗*^(*n* = 140)	184.3 ± 117.5^*∗*^(*n* = 48)	194.7 ± 124.0^*∗*^(*n* = 54)
Serum phosphate (mg/dL)	3.8 ± 0.5 (*n* = 208)	3.9 ± 0.6 (*n* = 127)	3.8 ± 0.6 (*n* = 156)	3.8 ± 0.4 (*n* = 76)	3.8 ± 0.6 (*n* = 53)
25(OH) vitamin D (ng/mL)	33.7 ± 12.7 (*n* = 178)	30.7 ± 10.2 (*n* = 124)	34.1 ± 10.5 (*n* = 142)	34.2 ± 10.8 (*n* = 62)	36.6 ± 12.7 (*n* = 57)
Serum magnesium (mg/dL)	2.1 ± 0.2 (*n* = 139)	1.9 ± 0.2^*∗*^(*n* = 85)	1.9 ± 0.2^*∗*^(*n* = 89)	1.9 ± 0.2^*∗*^(*n* = 53)	1.9 ± 0.2^*∗*^(*n* = 53)
Serum uric acid (mg/dL)	4.4 ± 1.0 (*n* = 73)	5.5 ± 1.1^*∗*^(*n* = 40)	5.3 ± 1.2^*∗*^(*n* = 54)	5.2 ± 1.4^*∗*^(*n* = 36)	4.9 ± 1.4^*∗*^(*n* = 19)

Mean ± SD. ^*∗*^Indicates significant differences vs. basal.

## Data Availability

Data available on request. The data are available on request through a data access committee, institutional review board, or the authors themselves to Lucas Brun (e-mail: lbrun@unr.edu.ar).
